# Siblings and Early Childhood Development: Evidence from a Population-Based Cohort in Preschoolers from Shanghai

**DOI:** 10.3390/ijerph19095739

**Published:** 2022-05-09

**Authors:** Saishuang Wu, Donglan Zhang, Xinyue Li, Jin Zhao, Xiaoning Sun, Lu Shi, Yuping Mao, Yunting Zhang, Fan Jiang

**Affiliations:** 1 Department of Developmental and Behavioral Pediatrics, National Children’s Medical Center, Shanghai Children’s Medical Center, School of Medicine, Shanghai Jiao Tong University, Shanghai 200127, China; suzannewu@sjtu.edu.cn (S.W.); zhaojin0610@hotmail.com (J.Z.); sunxn1005@163.com (X.S.); 2 Division of Health Services Research, Department of Foundations of Medicine, New York University Long Island School of Medicine, New York, NY 11501, USA; donglan.zhang@nyulangone.org; 3 School of Data Science, City University of Hong Kong, Hong Kong 999077, China; xinyueli@cityu.edu.hk; 4 Pediatric Translational Medicine Institution, Shanghai Children’s Medical Center, School of Medicine, Shanghai Jiao Tong University, Shanghai 200127, China; 5 Shanghai Center for Brain Science and Brain-Inspired Technology, Shanghai 201602, China; 6 Department of Public Health Science, College of Behavioral, Social and Health Science, Clemson University, Clemson, SC 29634, USA; lus@clemson.edu; 7 Department of Communication Studies, California State University Long Beach, Long Beach, CA 90840, USA; yuping.mao@csulb.edu; 8 Child Health Advocacy Institute, National Children’s Medical Center, Shanghai Children’s Medical Center, School of Medicine, Shanghai Jiao Tong University, Shanghai 200127, China

**Keywords:** early childhood development, sibling, psychosocial well-being, school readiness

## Abstract

(1) Background: The current study aims to investigate the association between the presence of a sibling and early childhood development (ECD). (2) Methods: Data were obtained from a large-scale population-based cohort in Shanghai. Children were followed from three to six years old. Based on birth order, the sample was divided into four groups: single child, younger child, elder child, and single-elder transfer (transfer from single-child to elder-child). Psychosocial well-being and school readiness were assessed with the total difficulties score from the Strengths and Difficulties Questionnaire (SDQ) and the overall development score from the early Human Capability Index (eHCI), respectively. A multilevel model was conducted to evaluate the main effect of each sibling group and the group × age interaction effect on psychosocial well-being and school readiness. (3) Results: Across all measures, children in the younger child group presented with lower psychosocial problems (β = −0.96, 95% CI: −1.44, −0.48, *p* < 0.001) and higher school readiness scores (β = 1.56, 95% CI: 0.61, 2.51, *p* = 0.001). No significant difference, or marginally significant difference, was found between the elder group and the single-child group. Compared to the single-child group, the single-elder transfer group presented with slower development on both psychosocial well-being (Age × Group: β = 0.37, 95% CI: 0.18, 0.56, *p* < 0.001) and school readiness (Age × Group: β = −0.75, 95% CI: −1.10, −0.40, *p* < 0.001). The sibling-ECD effects did not differ between children from families of low versus high socioeconomic status. (4) Conclusion: The current study suggested the presence of a sibling was not associated with worse development outcomes in general. Rather, children with an elder sibling are more likely to present with better ECD.

## 1. Introduction

Early childhood development (ECD) encompasses physical, socio-emotional, cognitive, and motor development of young children [1]. Closely aligned to the concept of ECD is the concept of school readiness, featured with qualities such as physical and nutritional well-being, intellectual skills, motivation to learn, and strong social-emotional capacity [2]. All these early capacities form the foundation for health and well-being in adulthood [3,4]. Advances in basic and intervention science indicate that ECD is particularly sensitive to complex and multi-faceted experiences. The most fundamental promotive early experiences come from the family environment [5,6,7]. According to key developmental theories, such as the family system theory and attachment theory [8,9,10], siblings are a fixture in the family lives of children and sibling experiences play critical roles in youth development and well-being. However, compared to the role of parenting, the sibling-ECD effect has been relatively understudied [11].

To date, results from studies on the role of siblings on ECD have been inconclusive. On the one hand, some studies concluded that increased family size has an adverse impact on the welfare of children, especially concerning their educational attainment. These studies mainly took a resource competition perspective and suggested that the presence of siblings reduced the investment on each child, as well as parents’ time and energy [12,13,14,15,16]. On the other hand, more recently, some researchers have recognized the value of peer companionships. In other words, with the importance of peer interaction in promoting socioemotional development being increasingly emphasized, siblings have been suggested as the closest, as well as the earliest, peers for young children. Corroborating with this perspective, empirical studies have reported that children benefit from a sibling companion in their developmental process [17]. That is, compared with children without siblings, those with siblings tend to have less internalizing and externalizing psychosocial problems [18,19,20]. Therefore, the role of siblings on ECD still needs further clarification.

Meanwhile, the sibling-ECD effects may be contingent on other factors. For instance, previous evidence has indicated that the age gap between siblings influenced the sibling effect on children’s developmental outcomes [21]. Socioeconomic status (SES), known as a strong indicator to ECD, may also impact the sibling effect through parenting style and family environment [22]. Therefore, it is worthwhile to further explore the sibling effect in different SES subgroups and different age gap subgroups. Additionally, other factors reported in previous studies, including sex, location of registered residency of the child, mother’s educational attainment, annual household income, primary caregiver, parental marital status, and parent-child interaction, likely all influence child’s birth order [23] and ECD [6,24,25], and so we included these factors as covariates in our analysis.

In the meantime, it shall be noted that the above-mentioned studies were conducted in western countries, yet the role of siblings is particularly worth studying in China. The different care-giving responsibilities of older siblings, the hierarchical structure of sibling roles, as well as cultural differences in dynamics, such as competition, have been noted between western and non-western societies [26]. Besides, there are special political factors that influence the family structure in China. Facing rapid population growth, China started the single-child policy in 1982. As effective as the policy was, new issues arose, including stagnant population growth, aging, and declining fertility rate. In response to these issues, in 2011, China announced that couples would be allowed to have two children, and then, in 2021, three children [27]. However, with social and economic patterns catering to the one-child policy, the promotion of fertility intention is particularly difficult in China [27]. Research has shown that policy factors may no longer be the major factors that affect the fertility intention of childbearing-age women [28]. Financial burden, mental stress, lack of family and social support, as well as concerns about not being able to provide the best possible environment for a child’s development are some of the most common reasons among parents for not having more than one child in China [29,30,31]. In short, it is unclear if the parents of the current generation are prepared for households with multiple children, which, in turn, makes it unclear how Chinese children of the current generation may be influenced by having a sibling or siblings. The current study utilized a population-based cohort in Shanghai, China, a metropolis with relatively high percentage of middle-class population, low fertility level, and low childbearing intentions [32], aiming to investigate the association between having a sibling and children’s early development, by measuring both school readiness and psychosocial well-being in children at the ages of three to six years.

## 2. Materials and Methods

### 2.1. Study Design and Participants

This study used data from a large-scale population-based survey, the Shanghai Children’s Health, Education and Lifestyle Evaluation-Preschool (SCHEDULE-P) [33]. The survey recruited newly enrolled kindergarteners in Shanghai, China, with a stratified cluster random sampling design in November 2016 (Entrance of kindergarten). The detailed sampling strategy and survey procedure was described elsewhere [33]. Children were followed up in April 2018 (Middle of kindergarten) and April 2019 (Graduation of kindergarten). The initial cohort sample included a total of 20,899 children aged 3–4 years from 191 kindergartens. Parents of 20,324 children (97.2%) consented to participate and completed the survey. All parents of children who participated in the study provided electronic informed consent at the beginning of the online survey.

### 2.2. Data Collection

#### 2.2.1. Sibling Groups

In the wave of Graduation, the caregiver was asked to answer “Is the child an only-child?”. If the response was “No”, the following question were asked: “How many other children do you have besides this child (show the child’s name)?” and birthday of each sibling of the child, and the answers were collected. Child number can be obtained from the two questions above. For each child who had a sibling, age gap was obtained by calculating the difference between the two children’s birthdays. Records were deleted if the age gap was biologically unreasonable (absolute value less than 7 months, *n* = 58). The birth order of each child in the Middle and Entrance waves was inferred by comparing the date of filling the questionnaire and the birthday of the sibling.

Based on single-child status and birth order, three groups were created in each wave: single-child (child who does not have a sibling), younger-child (the later-born child in the family), elder-child (the first-born child in the family). Families with more than two children were excluded due to very small sample size (*n* = 332). We excluded twins in the analysis because twin pregnancies are associated with a higher risk of preterm birth [34], a complication more likely to influence lag in development. Then children were divided into four groups based on the status of three waves: single (single-child in all three waves), younger (younger-child in all three waves), elder (elder-child in all three waves), single-elder transfer (transfer from single-child to elder-child).

#### 2.2.2. Early Childhood Development Measurement

We operationalized ECD in terms of psychosocial well-being and school readiness. We used the Strengths and Difficulties Questionnaire (SDQ) to measure psychosocial well-being. The SDQ identifies children with behavioral and emotional difficulties in clinical and community populations [35]. The questionnaire contains 25 attributes, each of which was scored as 0 = not true, 1 = somewhat true or 2 = certainly true. SDQ has five dimensions: emotional symptoms (e.g., many fears, easily scared), conduct problems (e.g., often lies or cheats), hyperactivity/inattention (e.g., restless, overactive, cannot stay still for long), peer relationship problems (e.g., gets on better with adults than with other children), and prosocial behavior (e.g., shares readily with other children [reverse scored]). The first four subscales were combined to generate a ‘total difficulties’ score indicative of psychosocial problems. The reliability has been tested by internal consistency. The Cronbach α coefficient is 0.59 for total difficulties [36].

The early Human Capability Index (eHCI) was used to measure school readiness. The eHCI is a population-level instrument designed to assess the development of children aged 36–59 months across diverse cultures [24,37,38]. There are a total of 62 survey questions in the eHCI reported by parents or caregivers. The eHCI contains aspects of human development [39] with 9 domains, which focuses more on children’s ability to cope with subsequent school life [38]. An overall development score is derived from all the 9 dimensions. A literacy and numeracy score, which measures the basic minimum skills and knowledge that are prerequisites for children to engage in learning activities, is derived from 3 of the 9 dimensions (numeracy and concepts, reading, and writing). The eHCI has been validated in 7 low- and middle-income countries, including China [38]. Our previous study on the Chinese version of eHCI indicated good internal reliability for both overall development (Cronbach’s α = 0.87) and literacy and numeracy (Cronbach’s α = 0.84). The test-retest reliability was 0.85, the inter-rater reliability between parents and teachers was 0.63. The majority of item factor loadings were above 0.7 and criterion-related validity compared with the Age and Stages Questionnaire was 0.53 [38]. Originally, scores for overall development and sub-domains were calculated on a 0-1 scale with higher scores indicating better school readiness [37]. For a more easily readable presentation of the results, we recoded all eHCI scores to a 0-100 scale by multiplying them by 100 in this article.

#### 2.2.3. Covariates

Demographic characteristics, including age, sex, and Hukou (the location of registered residency of the child, here we divided it into Shanghai or not Shanghai) of all participants were retrieved from the Shanghai Kindergarten Registry Database of the Shanghai Education Committee, and further confirmed by parents at the beginning of the survey. Parent’s educational attainment was categorized into 4 groups: high school or below, junior college, undergraduate, master or above. We included mother’s educational attainment (coded in Entrance) as a time invariant measure of socio-economic position. The annual household income was divided into 5 categories: <100 k Ren Min Bi (RMB), 100–150 k RMB, 150–300 k RMB, ≥300 k RMB and Unknown (Don’t know or Refused to answer). In Shanghai, households with annual income <100 k RMB were defined as low-income families who meet the requirement of applying for special assistance [40].

Family environment, including primary caregiver (parents or grandparents/others) was defined as the person(s) tasked with taking care of the child or children for the longest period in the past year and was reported by parents at each survey. Information on the parent’s marital status (Married/cohabitating or divorced/separated or Unknown) was also collected.

As parent-child interaction plays a key role in ECD, we also assessed it using the Chinese Parent-Child Interaction Scale (CPCIS), which was standardized and well-validated in the Chinese population [41]. The Chinese version of CPCIS indicated good internal reliability (Cronbach’s α = 0.82) and good person-separation reliability (0.81) [41]. The questionnaire measures the quantity of the most common four activities, including learning-related activities (e.g., “Teaching counting” and “Playing intellectual games together”), reading activities (e.g., “Reading together” and “Reading stories”), recreation activities (e.g., “Drawing together” and “Singing together”) and interaction with environment (e.g., “Discussing news and current affairs” and “Teaching about plants and animals”). The total CPCIS scores were calculated by adding all activity scores based on the frequency of each activity.

### 2.3. Statistical Analysis

As missing data in all variables were less than 5%, they were handled using listwise deletion. Descriptive analysis was conducted to summarize the characteristics of the overall sample and each group in Entrance. Locally weighted smoothing plots were used to describe the trend of total difficulties scores and overall development scores over age for each group, respectively.

We used a multilevel model to account for the longitudinal measurements of participants and evaluated the main effects of group and age, as well as the group × age interaction effect, on total difficulties scores and overall development scores, adjusting for sex, Hukou (location of registered residency of the child), mother’s educational attainment, annual household income, primary caregiver, parental marital status, and parent-child interaction. For each group, effects were estimated by both a main effect term (effect on ‘Initial status’, i.e., wave of Entrance) and an interaction term with time (effect on rate of change per year). To reduce the endogeneity induced by selection bias, we used inverse probability of weighting (IPW) using the propensity score [42] to balance the characteristics among groups in the multilevel model. Each child’s propensity score was estimated using a logistic regression model with a set of covariates (the child’s sex, Hukou, family annual income, mother’s educational attainment, parental marital status, and primary caregiver) that may help equalize the distribution of covariables among groups. Linear regression was then conducted to estimate the average effect [43] on psychosocial well-being and school readiness of each group compared with the single-child group, respectively.

The multilevel model was also used to evaluate the main effect of group and mother’s educational attainment (a proxy for socioeconomic status) and their interaction effects on total difficulties scores and overall development scores, adjusting for kindergarten education (Entrance of kindergarten or not), gender, age, Hukou (location of registered residency of the child), annual household income, primary caregiver, parental marital status, and parent-child interaction.

To further explore the effects of age gap between siblings, we divided the younger group into 4 groups according to the age gap between the child and his/her sibling: younger with age gap ≥9, younger with age gap 6 to 9, younger with age gap 3 to 6, Younger with age gap <3. There were no further groups for children in the elder group since the age gaps were mostly less than 3 years in Entrance. Then the multilevel models were used to evaluate the coefficients of each group on total difficulties scores and overall development scores compared with a single child.

All data analyses were conducted using Stata v 16.0 (StataCorp LP, College Station, TX, USA).

## 3. Results

### 3.1. Demographic Characteristics

**Figure 1:**
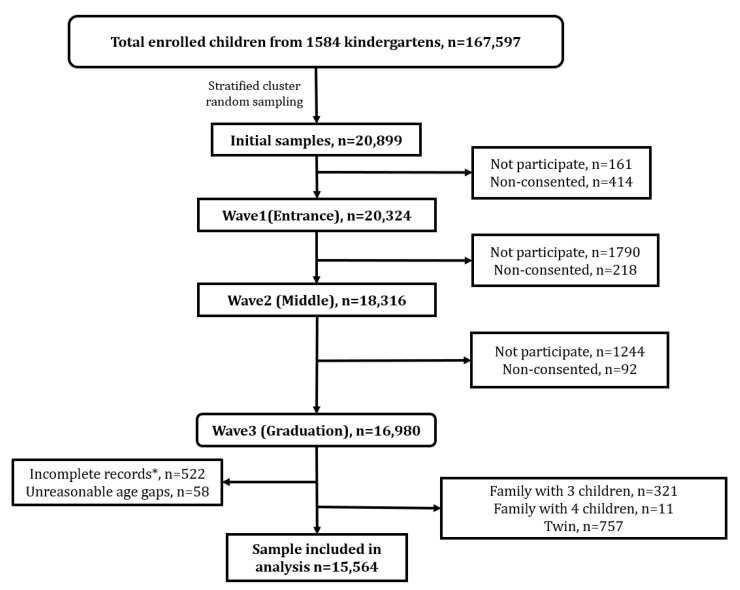
Flow chart of included analytical sample. * In any of the three waves, missing data on variables including SDQ, eHCI, age, sex, Hukou (the location of registered residency of the child), district, kindergarten classification, and CPCIS was considered as incomplete record.

Among the 20,324 children whose parents participated in the survey, 15,564 children had complete data for all three waves (three-year follow-up rate: 84.8%) and were taken as the final sample for further analyses (Figure 1). The characteristics of the participants are displayed in Table 1. The proportion of each group was: single (single-child in all three waves) 73.4% (*n* = 11,430), younger (younger-child in all three waves) 10.9% (*n* = 1698), elder (elder-child in all three waves) 7.2% (*n* = 1115), single-elder (transfer from single-child to elder-child) 8.5% (*n* = 1321). The mean age of the overall sample was 3.73 (SD = 0.29) during enrollment, 5.15 (SD = 0.29) in the first follow-up, 6.17 (SD = 0.29) in the second follow-up. Statistically significant differences among the 4 groups were found in sex, Hukou, mother’s educational attainment, annual household income (RMB), primary caregiver, and parental marital status.

### 3.2. Descriptive Results for Total Difficulties Score and Overall Development Score

**Figure 2:**
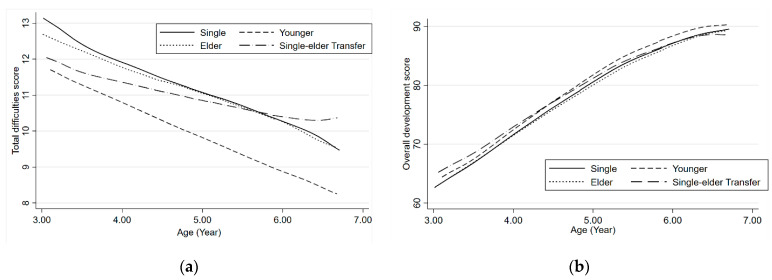
(**a**) Locally weighted smoothing plots for total difficulties scores by SDQ over age in the different children’s groups. (**b**) Locally weighted smoothing plots for overall development scores by eHCI over age in the different children’s groups. (Add annotations for single, younger, etc.).

Figure 2 showed locally weighted smoothing plots for total difficulties scores by SDQ and overall development scores by eHCI over age in the different children groups. Figures S1 and S2 showed the locally weighted smoothing plots for the subdomains of SDQ and eHCI over age for each group, respectively.

### 3.3. Association between Having a Sibling and ECD

Results from mixed effects models revealed that at Entrance of kindergarten, compared with children in the single group, children in the younger group (β = −0.96, 95% CI: −1.44, −0.48, *p* < 0.0001) and the single-elder transfer group (β = −0.81, 95% CI: −1.25, −0.37, *p* < 0.0001) had significantly lower total difficulties scores. For overall development scores, both the younger group (β = 1.56, 95% CI: 0.61, 2.51, *p* = 0.001) and the single-elder transfer group (β = 1.97, 95% CI: 1.08, 2.85, *p* < 0.0001) had significantly higher scores, compared with the single group. For children in the elder group, both total difficulties scores and overall development scores were not significantly different from children in the single group (Table 2).

For children in the single group, as they grew older by one year, the total difficulties scores were estimated to decrease by 0.64 (95% CI: −0.78, −0.49) and the overall development scores were estimated to increase by 6.07 (95% CI: 5.78, 6.35). As for the rate of change (per year), as children in the single-elder transfer group grew older by one year, the total difficulties scores were estimated to decrease by 0.64 − 0.37 = 0.27 and the overall development scores were estimated to increase by 6.07 − 0.75 = 5.32, both were significantly slower than children in the single group. For children in the elder group, overall development scores increased by 6.07 − 0.61 = 5.46 per year, significantly slower than for the single group (Table 2). For subdomains of SDQ and eHCI, similar results can be found in Table S1.

Children in the younger group had lower total difficulties scores and higher overall development scores regardless of mother’s educational attainment (Table S2), though not all differences were significant. All younger children, regardless of their age gap group, had lower total difficulties scores and higher overall development scores than children in the single group (Table S3), though not all differences were significant.

## 4. Discussion

The current study investigated the association between having a sibling and ECD in kindergarten children at the ages of three to six using a population-based cohort in Shanghai, China, as well as impact of child’s age, mother’s educational attainment, and the age gap between siblings. Across all measures, the presence of an elder sibling was associated with reduced psychosocial problems and increased school readiness scores, regardless of the age gaps between them and their elder siblings. No significant difference, or marginally significant difference, was found between the elder group and the single group. Transferring from single- to elder-child during the ages of three to six was associated with slower development in both psychosocial well-being and school readiness. The coefficients of association did not differ between children from low and high SES families. To the best of our knowledge, this study is among the first to dynamically examine the association of having a sibling with ECD among Chinese children in their early years.

Numerous studies have found that risk factors, including mother’s educational attainment, responsive caregiving and early stimulation [22,25,44,45,46], have large effect sizes on a child’s development. Interestingly, though children in the younger group had the lowest mother’s educational attainment and parent-child interaction frequency among all groups, we found that this group showed advantages in both school readiness measured by eHCI and psychosocial well-being measured by SDQ, compared with the single child group. A similar pattern of later-born advantages was found in previous studies both in UK and Japan [17,23]. Interactions among siblings provide contexts to develop social and emotional competencies, which are known as protective factors for mental health problems [47,48]. When facing stressful life events, the presence of older siblings may also buffer negative effects on younger children [49]. Therefore, the benefit for non-cognitive development that a younger child receives from the company of their elder siblings may counterbalance the negative effects of reduced parent-child interaction at this age. Meanwhile, it is also worth noting that one study, which investigated sibling effects on infants’ early development in rural families in western China, found that having siblings hinders children’s cognitive, language, motor, and social-emotional skills [50]. The difference between results from rural and urban areas may be explained by the different vulnerability of family to resource constraints. Compared with a family living in coastal cities, such as Shanghai, families from low-income areas are relatively more vulnerable to resource constraints [51].

Results from the elder and single-elder transfer groups both provided insights for the sibling effect of being the first-born child. Children in the single-elder transfer group experienced a family structure transition and became older siblings upon the birth of a baby sibling during 3–6 years of age. While there was no significant difference in total difficulties scores between the single group and the elder group at 3 years old, compared with children in the single group, total difficulties scores decreased significantly for children in the single-elder transfer group during the follow-up. Psychoanalytic theories emphasize the stressful nature of this transition for firstborn children, often citing it as one of the most traumatic experiences of early childhood [52]. There is also evidence suggesting that children would have a worse emotional state and increasing behavior problems after the birth of a sibling [53,54]. Similar results were found in China, which indicated increased psychological adaptation problems in first-born children within half a year of the second child’s birth [55]. Nonetheless, these changes may be limited to the short-term [56]. A recent study reviewed world-wide studies on the first-born’s adjustment following the birth of a sibling and concluded that such adjustment may not be as disruptive as traditionally presumed. That is, the first-born was likely to present with some disruption, some growth, or no change at all during the transition into being a sibling [56]. So, following the development of children in the single-elder transfer group and observing if such effects reduce over time may be an interesting future research direction. Both the elder group and the single-elder transfer group experienced slower increase in overall development scores than the single group children; however with very small coefficients (0.61 and 0.75, respectively), compared with the effect size of age (6.07).

This study has several limitations. First, families that choose to have a second child likely present with certain characteristics that relate to the development of their children, such as better development of the first-born, occupational factors of parents, and physical health of parents. In other words, the presence of selection bias is possible. Nevertheless, through sensitivity analyses, the IPW models corroborated the robustness of our findings. Second, all the outcomes were measured by parent-reported questionnaires, which possibly increased bias for our study. Nevertheless, we utilized instruments with good reliability and validity to minimize the bias. Third, our study focused on the preschool age, limiting our ability to estimate the sibling effect in the long-term. Future studies, with other age groups or further follow-ups, are strongly encouraged to clarify long-term sibling effects. Fourth, though Shanghai, a typical region in China with a continuously decreasing fertility rate, is ideal to serve the aims of this study to investigate parents’ concerns about the possible negative impact of having a second child on children’s development, we are aware that the study site could lead to limitation of generalization. Interestingly, similar results were found in western societies [17], which suggest that our findings might apply to various cultural and social contexts.

## 5. Conclusions

Overall, the current study revealed that, compared with single children, children with an elder sibling had better development outcomes, regardless of socioeconomic status, while children with a younger sibling were found to have no, or marginal, difference with a single child. In addition, children who experienced the transfer from single- to elder- child during the preschool period were found to have slower development. Our findings are, overall, consistent with those being found in both Western and non-Western countries, expect for those in poverty-stricken areas. Together, these results provide empirical evidence to dispel parents’ concerns that having more than one child will negatively affect each child’s development. Under the context of decreasing global fertility rates, empirical evidence from our results could help inform parents about the facts that having a second child is not negatively related to the development of their children. To address the global challenges posed by declining fertility rates, results of our study should be disseminated to the public and have a positive effect on the promotion of fertility intention.

## Figures and Tables

**Table 1:** Descriptive statistics of overall sample and each group in Entrance wave.

Characteristics	Mean (SD)/N (Proportion)					
	All Sample (*n* = 15,564)	Single (*n* = 11,430)	Younger (*n* = 1698)	Elder (*n* = 1115)	Single-Elder Transfer (*n* = 1321)	2-Tailed *p* Value ^2^
Age	3.73 (0.29)	3.73 (0.29)	3.71 (0.30)	3.77 (0.29)	3.72 (0.29)	<0.001
Sex						
Boy	8083 (51.9%)	6060 (53.0%)	921 (54.2%)	494 (44.3%)	608 (46.0%)	<0.001
Girl	7481 (48.1%)	5370 (47.0%)	777 (45.8%)	621 (55.7%)	713 (54.0%)	
Hukou ^1^						
Shanghai	12,657 (81.3%)	9756 (85.4%)	1120 (66.0%)	834 (74.8%)	947 (71.7%)	<0.001
Not Shanghai	2907 (18.7%)	1674 (14.7%)	578 (34.0%)	281 (25.2%)	374 (28.3%)	
Mother’s educational attainment						
High school or below	2381 (15.3%)	1501 (13.1%)	540 (31.8%)	162 (14.5%)	178 (13.5%)	<0.001
Junior college	3806 (24.5%)	2930 (25.6%)	380 (22.4%)	237 (21.3%)	259 (19.6%)	
Undergraduate	7595 (48.8%)	5768 (50.5%)	617 (36.3%)	550 (49.3%)	660 (50.0%)	
Master or above	1741 (11.2%)	1202 (10.5%)	153 (9.0%)	164 (14.7%)	222 (16.8%)	
Unknown or refused to answer	41 (0.3%)	29 (0.3%)	8 (0.5%)	2 (0.2%)	2 (0.2%)	
Annual Household Income (RMB)						
<100 k	2806 (18.0%)	2079 (18.2%)	370 (21.8%)	164 (14.7%)	193 (14.6%)	<0.001
100~150 k	2635 (16.9%)	2007 (17.6%)	259 (15.3%)	183 (16.4%)	186 (14.1%)	
150~300 k	5434 (34.9%)	4121 (36.1%)	477 (28.1%)	359 (32.2%)	477 (36.1%)	
≥300 k	3746 (24.1%)	2535 (22.2%)	471 (27.7%)	360 (32.3%)	380 (28.8%)	
Unknown or refused to answer	943 (6.1%)	688 (6.0%)	121 (7.1%)	49 (4.4%)	85 (6.4%)	
Primary caregiver						
Parents	9334 (60.0%)	6510 (57.0%)	1295 (76.3%)	716 (64.2%)	813 (61.5%)	<0.001
Grandparents/others	6230 (40.0%)	4920 (43.0%)	403 (23.7%)	399 (35.8%)	508 (38.5%)	
Parental marital status						
Married/cohabitating	14,828 (95.3%)	10,816 (94.6%)	1640 (96.6%)	1089 (97.7%)	1283 (97.1%)	<0.001
Divorced/separated	454 (2.9%)	369 (3.2%)	38 (2.2%)	14 (1.3%)	33 (2.5%)	
Unknown or refused to answer	282 (1.8%)	245 (2.1%)	20 (1.2%)	12 (1.1%)	5 (0.4%)	
CPCIS score	2.80 (0.95)	2.83 (0.95)	2.56 (0.98)	2.80 (0.94)	2.86 (0.93)	<0.001

^1^ Hukou represent the location of registered residency of the child. Here we divided it into Shanghai (local) or not Shanghai (nonlocal). Abbreviations: RMB, Ren Min Bi; CPCIS, the Chinese Parent-Child Interaction Scale. ^2^ Chi-square test or ANOVA were conducted to test the difference across the groups (single, younger, elder, single-elder) for each characteristic.

**Table 2:** Association between birth order and early childhood development.

	Total Difficulties Score		Overall Development Score	
	Adjusted *β* (95% CI)	2-Tailed *p* Value	Adjusted *β* (95% CI)	2-Tailed *p* Value
Initial status (at Entrance)				
Single	Ref.		Ref.	
Younger	−0.96 (−1.44, −0.48)	<0.001	1.56 (0.61, 2.51)	0.001
Elder	−0.06 (−0.51, 0.40)	0.798	0.90 (−0.16, 1.96)	0.097
Single-elder transfer	−0.81 (−1.25, −0.37)	<0.001	1.97 (1.08, 2.85)	<0.001
Age	−0.64 (−0.78, −0.49)	<0.001	6.07 (5.78, 6.35)	<0.001
Rate of change (per year)				
Single	Ref.		Ref.	
Younger	−0.19 (−0.39, 0.00)	0.055	0.01 (−0.36, 0.38)	0.963
Elder	0.05 (−0.15, 0.25)	0.630	−0.61 (−1.05, −0.17)	0.007
Single-elder transfer	0.37 (0.18, 0.56)	<0.001	−0.75 (−1.10, −0.40)	<0.001

Note: Age was subtracted by 3 to facilitate the interpretation of the coefficients. Adjusting for selected characteristics including the child’s sex, Hukou, family annual income, mother’s educational attainment, parental marital status, and primary caregiver that may which may help equalize the distribution of covariables among groups.

## Data Availability

Not applicable.

## Supplementary Materials

The following supporting information can be downloaded at: https://www.mdpi.com/article/10.3390/ijerph19095739/s1, Figure S1: Locally weighted smoothing plots for SDQ subdomains over age in different children groups. (add annotations for single, younger, etc.); Figure S2: Locally weighted smoothing plots for eHCI subdomains over age in different children groups. (add annotations for single, younger, etc.); Table S1: Mixed effects model results for subdomains of SDQ and eHCI in each children group compared with single group, Table S2: Association between birth order and early childhood development among different SES status, Table S3: Association between sibling age gaps and children’s early childhood development.
