# Supplementary material for: Siblings and Early Childhood Development: Evidence from a Population-Based Cohort in Preschoolers from Shanghai
Source: Int J Environ Res Public Health. 2022 May 9;19(9):5739. doi: 10.3390/ijerph19095739 (PMC9099463; doi:10.3390/ijerph19095739)
Supplement: Supplementary file 1 [file ijerph-19-05739-s001.zip › ijerph-1710853-supplementary.pdf]

Supplementary Material

Figure S1. Locally weighted smoothing plots for SDQ subdomains over age in different children groups. (add annotations for single, younger, etc.)

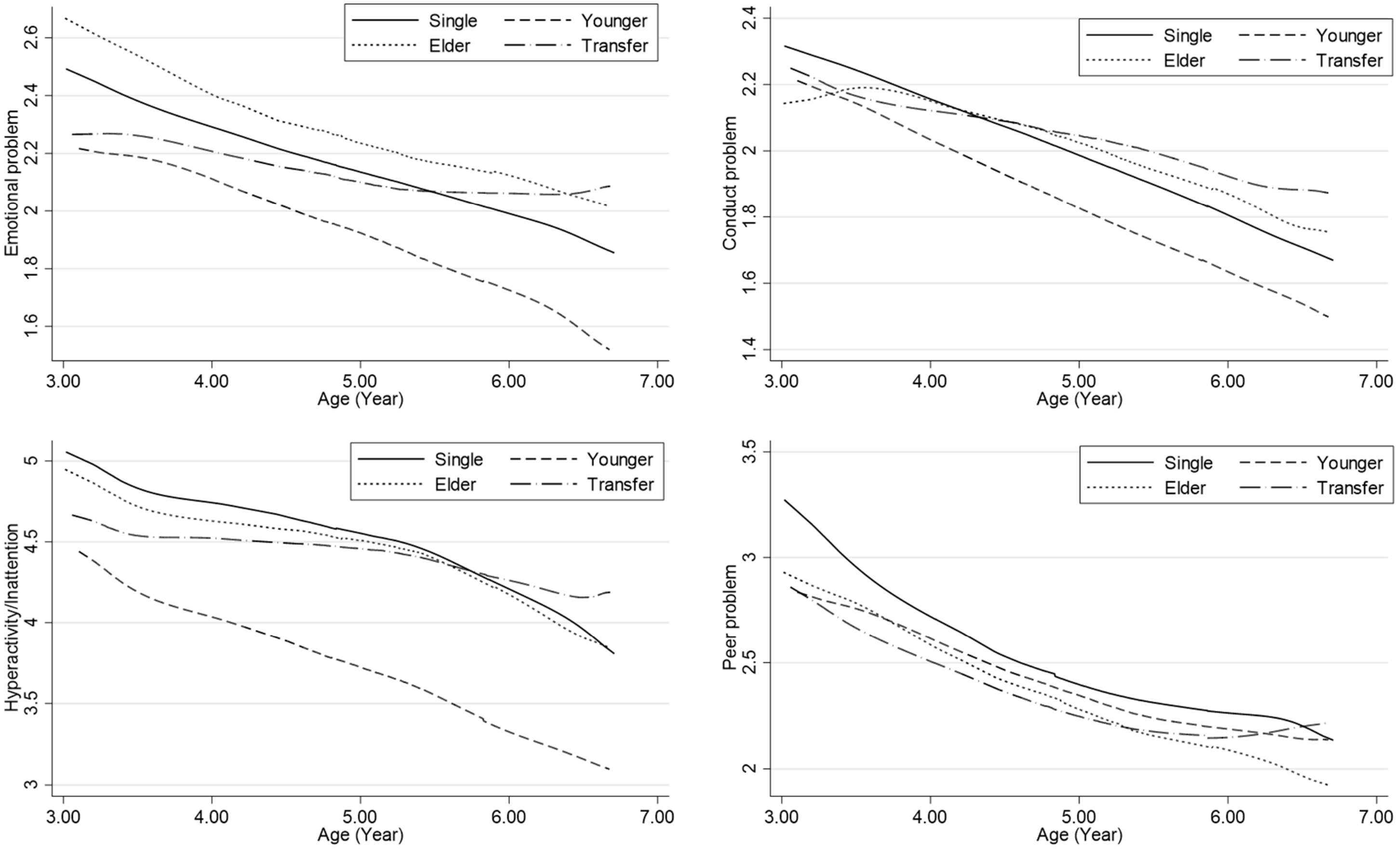

Transfer: Single-elder transfer group

Figure S2. Locally weighted smoothing plots for eHCI subdomains over age in different children groups. (add annotations for single, younger, etc.)

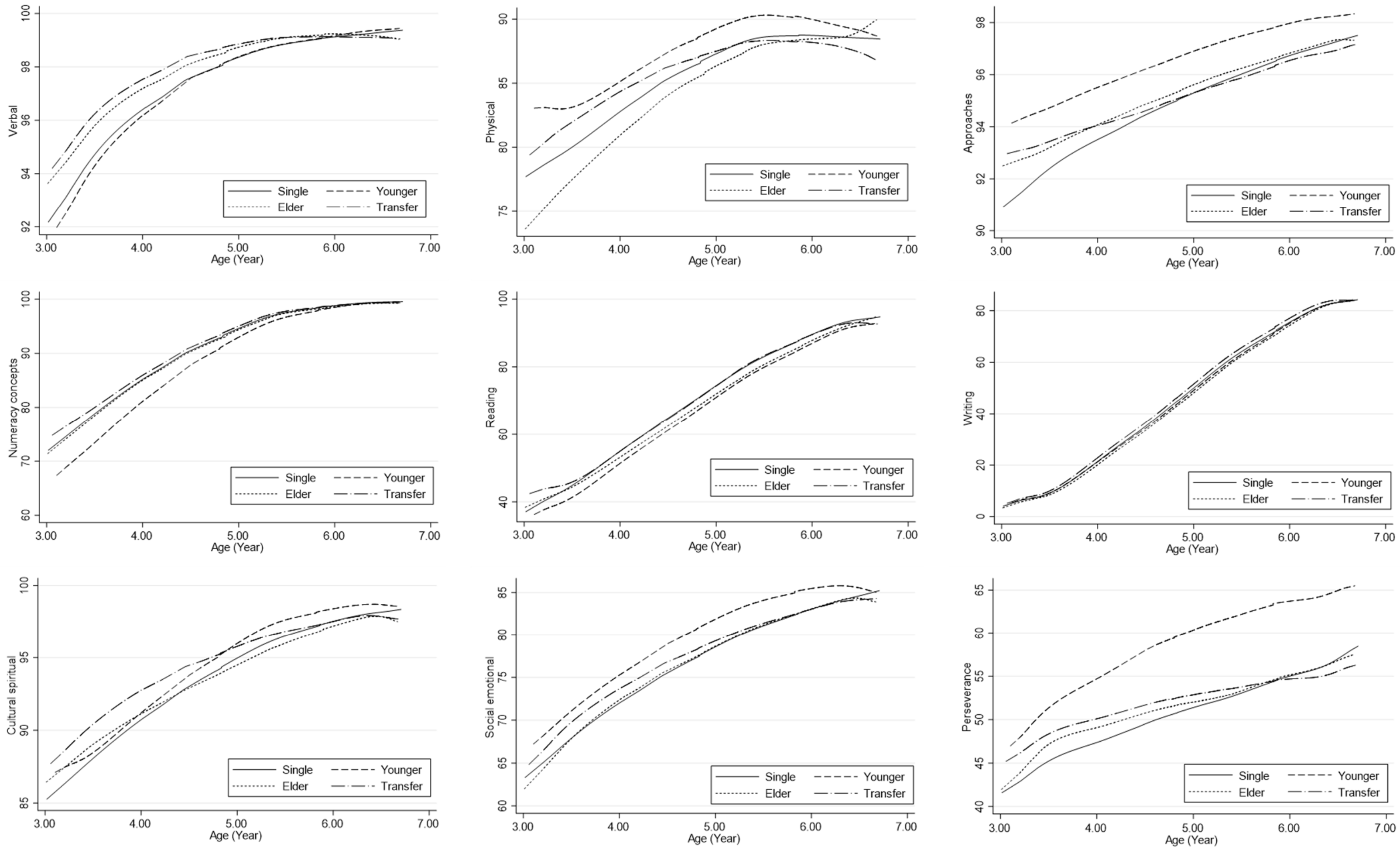

Transfer: Single-elder transfer group

Table S1. Mixed effects model results for subdomains of SDQ and eHCI in each children group compared with single group.

		Younger		Elder		Single-elder transfer	
		Adjusted β (95% CI)	2-Tailed P value	Adjusted β (95% CI)	2-Tailed P value	Adjusted β (95% CI)	2-Tailed P value
**SDQ**							
Emotional symptoms	Initial status	-0.10 (-0.28, 0.09)	0.306	0.06 (-0.13, 0.24)	0.556	-0.12 (-0.29, 0.06)	0.192
	Rate of change (per year)	-0.06 (-0.14, 0.01)	0.111	0.03 (-0.05, 0.11)	0.418	0.06 (-0.02, 0.14)	0.119
Conduct problems	Initial status	-0.11 (-0.25, 0.03)	0.133	-0.06 (-0.20, 0.08)	0.386	-0.10 (-0.23, 0.03)	0.122
	Rate of change (per year)	-0.03 (-0.09, 0.03)	0.388	0.05 (-0.01, 0.11)	0.126	0.08 (0.02, 0.14)	0.007
Hyperactivity/inattention	Initial status	-0.66 (-0.85, -0.48)	<.001	-0.03 (-0.23, 0.18)	0.796	-0.40 (-0.59, -0.22)	<.001
	Rate of change (per year)	-0.09 (-0.17, -0.02)	0.017	0.01 (-0.08, 0.09)	0.870	0.18 (0.10, 0.26)	<.001
Peer relationship	Initial status	-0.10 (-0.24, 0.05)	0.181	-0.03 (-0.19, 0.14)	0.758	-0.19 (-0.34, -0.03)	0.020
	Rate of change (per year)	-0.01 (-0.07, 0.05)	0.707	-0.04 (-0.11, 0.03)	0.266	0.04 (-0.02, 0.11)	0.213
**eHCI**							
Verbal	Initial status	0.55 (-0.52, 1.62)	0.315	2.21 (1.17, 3.25)	<.001	2.47 (1.56, 3.38)	<.001
	Rate of change (per year)	-0.25 (-0.68, 0.18)	0.248	-0.86 (-1.30, -0.42)	<.001	-0.95 (-1.34, -0.56)	<.001
Physical	Initial status	4.00 (2.04, 5.96)	<.001	-0.88 (-3.23, 1.47)	0.465	2.40 (0.44, 4.36)	0.017
	Rate of change (per year)	-1.10 (-1.91, -0.30)	0.007	-0.06 (-1.05, 0.93)	0.902	-1.10 (-1.94, -0.27)	0.010
Approaches	Initial status	2.73 (1.53, 3.94)	<.001	1.39 (-0.14, 2.91)	0.076	1.96 (0.75, 3.18)	0.001
	Rate of change (per year)	-0.60 (-1.11, -0.09)	0.022	-0.54 (-1.21, 0.12)	0.109	-0.95 (-1.47, -0.42)	<.001
Numeracy Concepts	Initial status	-3.84 (-5.28, -2.39)	<.001	1.55 (-0.02, 3.12)	0.053	2.00 (0.66, 3.34)	0.003
	Rate of change (per year)	1.49 (0.93, 2.06)	<.001	-0.67 (-1.31, -0.03)	0.041	-0.76 (-1.31, -0.20)	0.007
Reading	Initial status	-2.60 (-4.59, -0.60)	0.011	0.18 (-2.12, 2.49)	0.877	0.51 (-1.67, 2.68)	0.648

	Rate of change (per year)	0.71 (-0.06, 1.48)	0.069	-0.78 (-1.68, 0.12)	0.091	-0.12 (-0.97, 0.72)	0.774
Writing	Initial status	2.52 (0.32, 4.72)	0.025	-1.99 (-4.38, 0.40)	0.103	-0.88 (-2.98, 1.21)	0.408
	Rate of change (per year)	-0.85 (-1.83, 0.13)	0.090	0.07 (-0.98, 1.13)	0.891	0.74 (-0.19, 1.66)	0.119
Cultural spiritual	Initial status	0.79 (-0.87, 2.46)	0.350	1.88 (-0.07, 3.82)	0.058	2.87 (1.44, 4.30)	<.001
	Rate of change (per year)	0.18 (-0.51, 0.87)	0.608	-1.03 (-1.86, -0.21)	0.014	-1.07 (-1.65, -0.49)	<.001
Social emotional	Initial status	3.71 (1.94, 5.48)	<.001	0.83 (-1.20, 2.86)	0.425	2.56 (0.84, 4.28)	0.004
	Rate of change (per year)	-0.42 (-1.12, 0.28)	0.238	-0.49 (-1.33, 0.35)	0.257	-1.07 (-1.76, -0.38)	0.002
Perseverance	Initial status	6.36 (3.63, 9.09)	<.001	2.71 (-0.23, 5.65)	0.071	3.52 (1.04, 6.01)	0.005
	Rate of change (per year)	0.93 (-0.20, 2.06)	0.108	-1.09 (-2.32, 0.13)	0.081	-1.43 (-2.47, -0.40)	0.007

Adjusting for potential confounders including kindergarten education, age, gender, Hukou (location of registered residency of the child), mother's educational levels, annual household income, primary caregiver, parental marital status, and parent-child interaction.

Table S2. Association between birth order and early childhood development among different SES status.

	Total difficulties score		Overall development score	
	Adjusted β (95% CI)	2-Tailed P value	Adjusted β (95% CI)	2-Tailed P value
**High school or below (n=2,381)**				
Initial status (at Entrance)				
Single	Ref.		Ref.	
Younger	-0.23 (-1.14, 0.68)	0.615	4.56 (2.30, 6.82)	<.001
Elder	0.03 (-1.26, 1.32)	0.961	2.71 (-1.20, 6.62)	0.174
Single-elder transfer	0.25 (-1.44, 1.94)	0.773	4.84 (2.00, 7.69)	0.001
Age	-0.45 (-0.91, 0.01)	0.056	6.96 (5.99, 7.94)	<.001
Rate of change (per year)				
Single	Ref.		Ref.	
Younger	-0.37 (-0.76, 0.01)	0.059	-1.24 (-2.17, -0.32)	0.008
Elder	0.17 (-0.38, 0.71)	0.548	-1.52 (-3.15, 0.11)	0.068
Single-elder transfer	0.16 (-0.61, 0.93)	0.690	-2.13 (-3.30, -0.96)	<.001
**Junior college (n=3,806)**				
Initial status (at Entrance)				
Single	Ref.		Ref.	
Younger	-1.40 (-2.28, -0.51)	0.002	1.85 (0.07, 3.63)	0.041
Elder	0.78 (-0.03, 1.58)	0.058	-1.14 (-3.20, 0.93)	0.281
Single-elder transfer	-0.68 (-1.62, 0.26)	0.155	0.66 (-1.06, 2.37)	0.454
Age	-0.93 (-1.18, -0.68)	<.001	6.49 (6.06, 6.92)	<.001
Rate of change (per year)				
Single	Ref.		Ref.	
Younger	-0.13 (-0.53, 0.27)	0.533	-0.05 (-0.77, 0.66)	0.882
Elder	-0.20 (-0.53, 0.14)	0.249	-0.12 (-0.95, 0.72)	0.785

Single-elder transfer	0.22 (-0.18, 0.63)	0.282	-0.14 (-0.77, 0.50)	0.677
**Undergraduate (n=7,595)**				
Initial status (at Entrance)				
Single	Ref.		Ref.	
Younger	-1.56 (-2.23, -0.89)	<.001	0.43 (-0.83, 1.69)	0.502
Elder	-0.38 (-1.04, 0.29)	0.267	0.62 (-0.74, 1.99)	0.369
Single-elder transfer	-0.88 (-1.41, -0.35)	0.001	0.81 (-0.34, 1.97)	0.169
Age	-0.59 (-0.77, -0.41)	<.001	5.51 (5.18, 5.84)	<.001
Rate of change (per year)				
Single	Ref.		Ref.	
Younger	-0.10 (-0.40, 0.20)	0.514	0.61 (0.12, 1.09)	0.015
Elder	0.26 (-0.04, 0.56)	0.085	-0.51 (-1.06, 0.05)	0.075
Single-elder transfer	0.47 (0.24, 0.71)	<.001	-0.43 (-0.87, 0.01)	0.056
**Master or above (n=1,741)**				
Initial status (at Entrance)				
Single	Ref.		Ref.	
Younger	-0.91 (-2.76, 0.95)	0.338	3.19 (0.67, 5.72)	0.013
Elder	0.26 (-0.82, 1.34)	0.633	0.44 (-1.95, 2.84)	0.717
Single-elder transfer	-0.80 (-1.82, 0.23)	0.127	1.84 (-0.30, 3.97)	0.091
Age	-0.53 (-0.89, -0.17)	0.004	5.54 (4.91, 6.17)	<.001
Rate of change (per year)				
Single	Ref.		Ref.	
Younger	-0.43 (-1.26, 0.40)	0.311	-0.52 (-1.60, 0.55)	0.341
Elder	-0.41 (-0.88, 0.06)	0.087	0.07 (-0.90, 1.04)	0.894
Single-elder transfer	0.36 (-0.10, 0.82)	0.129	-0.43 (-1.29, 0.44)	0.332

Adjusting for potential confounders including kindergarten education, gender, Hukou (location of registered residency of the child), annual household income, primary caregiver, parental marital status, and parent-child interaction.

Table S3. Association between sibling age gaps and children's early childhood development.

	Total difficulties score		Overall development score	
	Adjusted β (95% CI)	2-Tailed P value	Adjusted β (95% CI)	2-Tailed P value
Initial status (at Entrance)				
Single	Ref.		Ref.	
Younger with age gap ≥9y	-0.12 (-0.98, 0.75)	0.790	2.04 (0.00, 4.09)	0.050
Younger with age gap 6y to 9y	-1.73 (-2.77, -0.70)	0.001	1.68 (-0.28, 3.63)	0.093
Younger with age gap 3y to 6y	-1.07 (-1.78, -0.37)	0.003	1.62 (0.22, 3.02)	0.024
Younger with age gap <3y	-0.75 (-1.58, 0.09)	0.081	1.10 (-0.45, 2.65)	0.164
Elder	-0.06 (-0.51, 0.40)	0.798	0.90 (-0.16, 1.96)	0.097
Single-elder transfer	-0.81 (-1.25, -0.37)	<.001	1.97 (1.08, 2.85)	<.001
Age	-0.64 (-0.78, -0.49)	<.001	6.07 (5.78, 6.35)	<.001
Rate of change (per year)				
Single	Ref.		Ref.	
Younger with age gap ≥9y	-0.36 (-0.66, -0.05)	0.021	-0.43 (-1.20, 0.34)	0.271
Younger with age gap 6y to 9y	0.15 (-0.31, 0.61)	0.534	-0.03 (-0.79, 0.74)	0.943
Younger with age gap 3y to 6y	-0.21 (-0.53, 0.10)	0.186	0.14 (-0.46, 0.73)	0.654
Younger with age gap <3y	-0.31 (-0.68, 0.05)	0.092	0.13 (-0.45, 0.71)	0.663
Elder	0.05 (-0.15, 0.25)	0.630	-0.61 (-1.05, -0.17)	0.007
Single-elder transfer	0.37 (0.18, 0.56)	<.001	-0.75 (-1.10, -0.40)	<.001

Adjusting for potential confounders including kindergarten education, age, gender, Hukou (location of registered residency of the child), mother's educational levels, annual household income, primary caregiver, parental marital status, and parent-child interaction.
